# Advances in the regulation of adipogenesis and lipid metabolism by exosomal ncRNAs and their role in related metabolic diseases

**DOI:** 10.3389/fcell.2023.1173904

**Published:** 2023-09-18

**Authors:** Cong Liu, Xilin Liu, Hong Li, Zhichen Kang

**Affiliations:** ^1^ Department of Breast Surgery, China-Japan Union Hospital of Jilin University, Changchun, China; ^2^ Department of Hand and Foot Surgery, China-Japan Union Hospital of Jilin University, Changchun, China; ^3^ Department of Nursing, China-Japan Union Hospital of Jilin University, Changchun, China; ^4^ Department of Rehabilitation, The Second Hospital of Jilin University, Changchun, China

**Keywords:** exosomes, ncRNA, adipogenesis, lipid metabolism, obesity-related metabolic diseases

## Abstract

Exosomes are membrane-bound extracellular vesicles released following the fusion of multivesicular bodies (MVBs) with the cell membrane. Exosomes transport diverse molecules, including proteins, lipids, DNA and RNA, and regulate distant intercellular communication. Noncoding RNA (ncRNAs) carried by exosomes regulate cell-cell communication in tissues, including adipose tissue. This review summarizes the action mechanisms of ncRNAs carried by exosomes on adipocyte differentiation and modulation of adipogenesis by exosomal ncRNAs. This study aims to provide valuable insights for developing novel therapeutics.

## 1 Introduction

Non-coding RNAs (ncRNAs) are RNA molecules that do not encode proteins. ncRNAs act as regulators of gene expression at various levels, including the transcriptional, post-transcriptional and translational levels. ncRNAs include micro-RNAs (miRNAs), long-chain non-coding RNAs (lncRNAs), and circular RNAs (circRNAs) (Kapranov et al., 2007). miRNAs are highly conserved small ncRNAs (Holoch and Moazed, 2015). On the other hand, lncRNAs are poorly conserved ncRNAs with a length above 200 nt. LncRNAs act as competitive endogenous RNA (ceRNA) by binding to complementary binding sites on miRNA, thus regulating target gene expression by binding to the 3′UTR region (He et al., 2020). circRNAs are highly conserved ncRNAs with a circular structure formed by covalent binding of the 3′ end and the 5′ end after back-splicing. circRNAs also regulate gene and protein expression by serving as miRNA and RNA-binding protein sponges (Aufiero et al., 2019). ncRNAs are associated with various diseases and have diagnostic and therapeutic value (Wang et al., 2021). Genomic DNA can be transcripted into coding RNA or non-coding RNA. Approximately two percent of the human genome is made up of protein-coding genes (Kaikkonen and Adelman, 2018). Non-coding RNAs are not translated into proteins (Adams et al., 2017). Non-coding RNAs can be divided into long-chain noncoding rNA (lncRNAs, >200 nucleotides), medium-chain ncRNAs (20-200 nucleotides), and short-chain ncRNAs (<20 nucleotides). Short-chain ncRNAs can be further divided into piwi-interacting RNA, small interfering RNAs (siRNAs), and miRNAs. Medium-chain ncRNAs include small-nuclear RNA involved in transcript splicing in protein synthesis, nucleolar RNA involved in ribosomal RNA modification, transcription start site (TSS)-related RNA, and promoter-related miRNA. miRNAs play an important regulatory role at different stages of lipid metabolism (Aryal et al., 2017).

Studies have demonstrated that lncRNAs regulate various physiological and pathological processes, including growth and development, hematopoietic processes, cell proliferation and apoptosis, tumorigenesis, cell metabolism, genomic imprinting, chromatin modification, infection, and immune response (Karreth et al., 2015; Wang et al., 2016; Kasagi et al., 2017). lncRNAs interact with the target protein-coding genes through a highly complicated RNA regulatory network. Mutations or abnormal expressions of lncRNAs are closely linked to various diseases.

Valadi et al. demonstrated that exosomes could transport miRNAs and mRNAs into other cells (Valadi et al., 2007). Furthermore, miRNAs, lncRNAs, and circRNAs were shown to be encapsulated and transported intercellularly. When exosome ncRNAs undergo tissue-specific changes due to stimulation from various internal and external factors, they can cause organ dysfunction, aging, and disease. Previous studies have shown that exosomal ncRNAs participate in the pathogenesis and development of infectious, autoimmune, metabolic, neurodegenerative, cardiovascular, and neurodegenerative diseases. Exosomal ncRNAs also show differential expression in different cells or under different physiological or pathological conditions. Therefore, exosome ncRNAs serve as potential diagnostic and therapeutic biomarkers.

On the other hand, different biological processes, such as differentiation, proliferation, apoptosis, metabolism, immune responses, and tumorigenesis, are influenced by miRNAs (Alvarez-Garcia and Miska, 2005; Schickel et al., 2008). Yan et al. (2016) showed differential expression of miRNAs between the plasma of patients with diabetes and controls. miRNAs are differentially expressed in different organs related to metabolism, including the liver, pancreatic islets, and adipose tissue. In recent years, significant advances have been made in understanding the regulation of adipocyte differentiation (Esau et al., 2004; Kajimoto et al., 2006; Cheung et al., 2008; Lin et al., 2009). Several protein-coding genes, mRNAs, and microRNAs are associated with lipid metabolism and adipocyte differentiation.

However, gene expression profiles and functions of lncRNAs during adipocyte differentiation remain to be fully analyzed. Sun et al. (2013) identified 175 lncRNAs which were significantly dysregulated during adipocyte differentiation by profiling transcriptomes of primary brown and white adipocytes, precursor adipocytes and mature adipocytes. They found that C/EBPα and PPARγ could bind to the promoter regions of many lncRNAs. It was also revealed that inhibition of ten lncRNAs by RNAi significantly inhibited lipid droplet formation. Few studies have investigated the expression profile of non-coding RNAs in human adipose-derived stem cells (hADSCs). The regulatory mechanisms and pathways of adipocyte differentiation by ncRNAs remain unstudied. A recent study revealed that adipose tissue-derived exosomes could regulate physiological signal transduction and metabolism in adipose tissue and other peripheral tissues (Wang et al., 2022). Adipose tissue-derived exosomes could contribute to the development of obesity and related metabolic syndromes. Adipose tissue can regulate the metabolic homeostasis of various tissues and organs by secreting exosomes (Mathieu et al., 2019). Furthermore, exosomes derived from various tissues can disproportionately affect lipid accumulation and metabolism in adipose tissue. Previous studies demonstrated that ncRNAs regulate cell differentiation, epigenetics, and cell cycle regulation. In addition, ncRNAs can modulate adipocyte differentiation and adipogenesis by targeting transcription factors and signaling molecules. In this review, we outline the exosome production pathways and the molecular regulatory mechanisms of ncRNAs carried by adipogenic exosomes. The review aims to provide valuable insights for treating metabolic diseases by targeting exosome production.

## 2 Exosomes in regulating adipocyte differentiation and lipid metabolism

**FIGURE 1:**
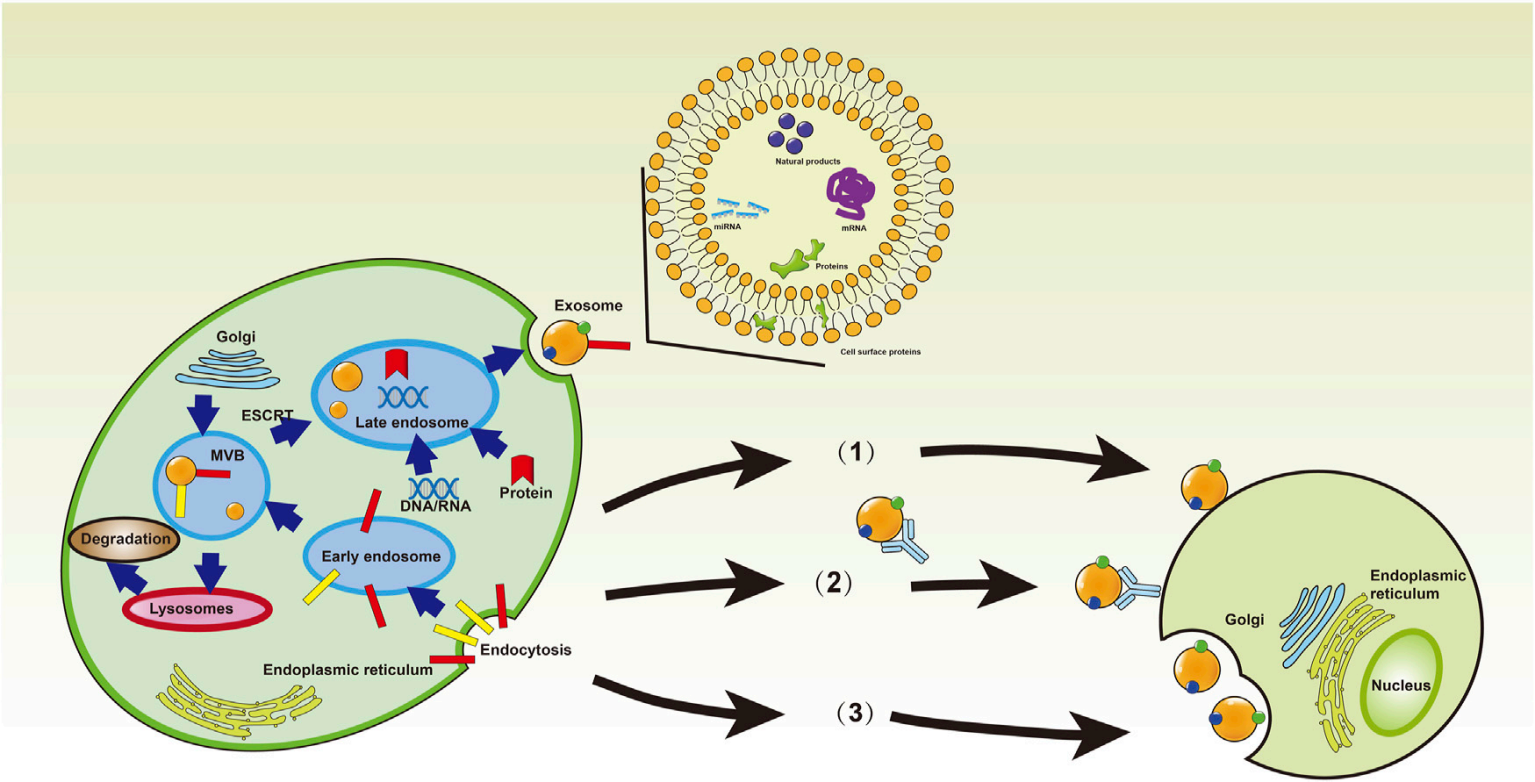
The forming process and structure of the exosomes. Donor cells secrete exosomes into the extracellular space, and exosomes can carry various cargos and interact with recipient cells through endocytosis, or direct membrane fusion, or receptor-ligand interfaces [Reprinted by permission from John Wiley and Sons Ltd. Journal of cellular and molecular medicine, Xing et al. (2021), copyright 2021].

**FIGURE 2:**
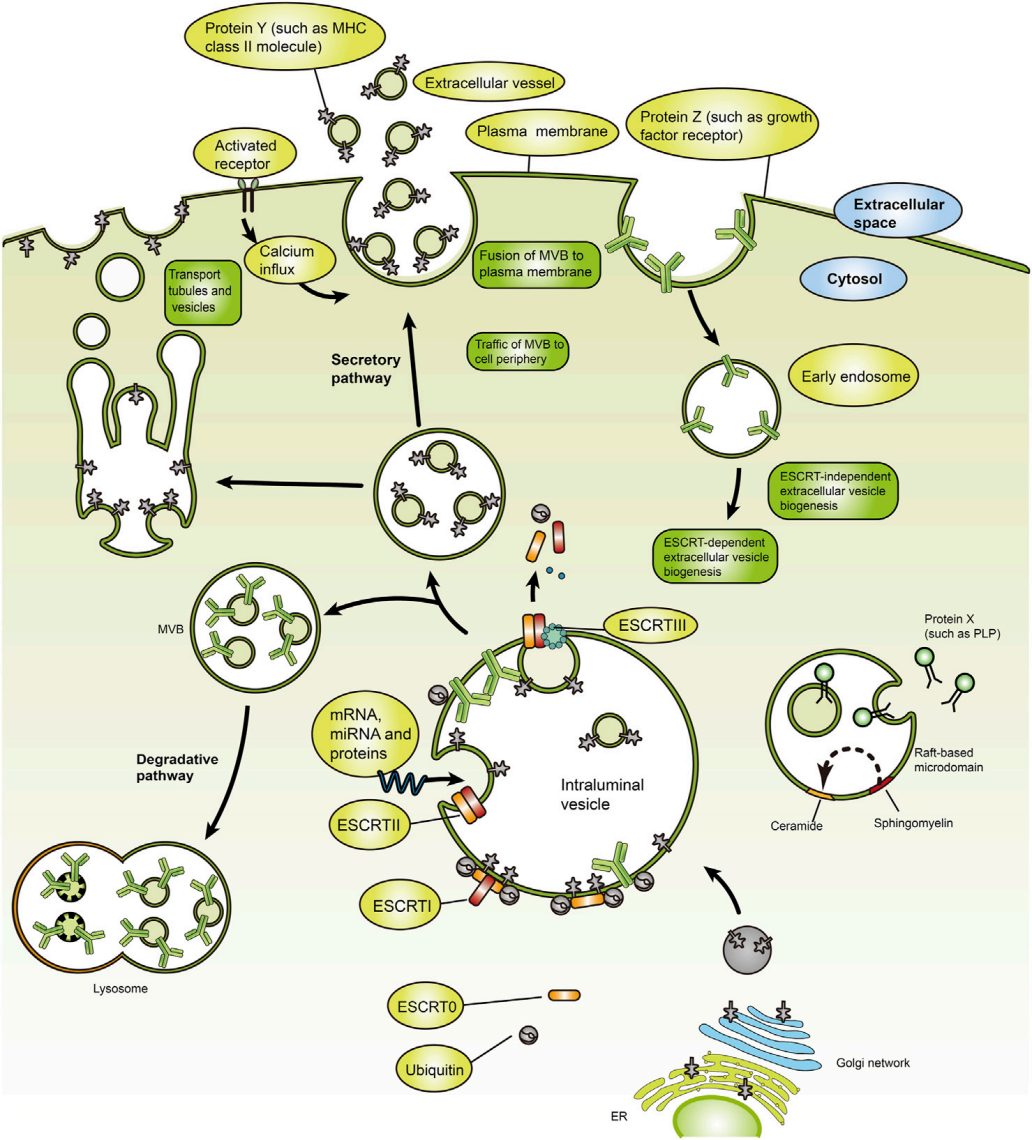
Mechanisms for biogenesis and secretion of exosomes. Exosome-producing MVBs travel along the secretory pathway, move to the cell periphery, fuse with the plasma membrane, and release their ILVs into the extracellular environment. MVBs can interact dynamically with other organelles or compartments. The exosome formation from MVBs proceeds via ESCRT-dependent and ESCRT-independent pathways [Reprinted by permission from Macmillan Publishers Ltd. Nature Reviews Immunology, Robbins and Morelli (2014), copyright 2014].

**FIGURE 3:**
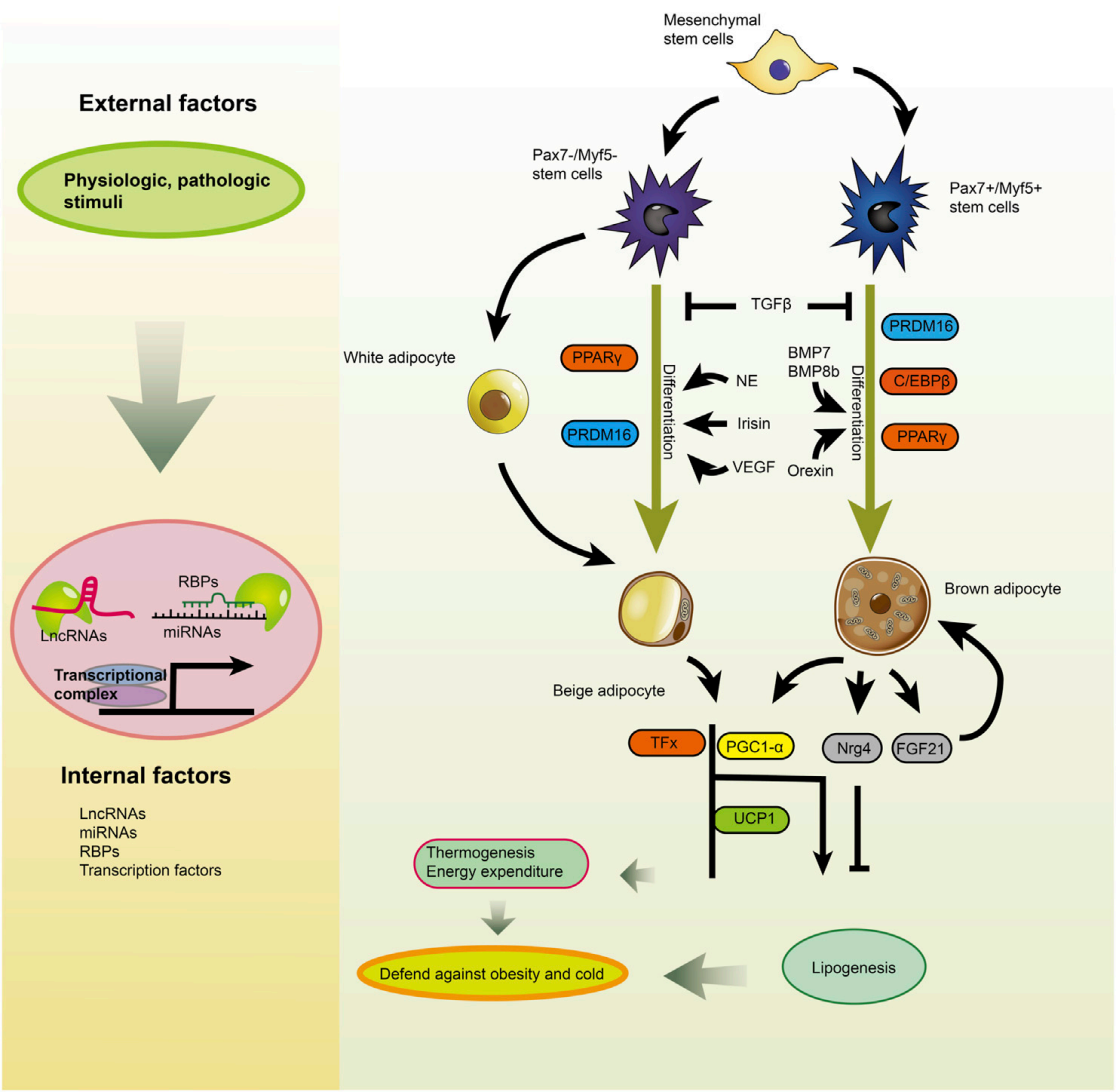
Global protein/ncRNA regulatory networks during brown/beige adipogenesis. Brown and beige adipocytes have different progenitors and are affected differently either by intrinsic factors or extrinsic factors that indirectly regulate key transcription factors [Reprinted by permission from Elsevier Inc. Advances in clinical chemistry, Zeng et al. (2018), copyright 2018].

After fusion of intracellular multivesicular bodies (MVBs) with the cell membrane, exosomes are discharged into the extracellular environment (Simpson et al., 2009; Mathivanan et al., 2010). Exosomes are crucial to maintaining human health. Therefore, understanding their role in diseases can provide valuable insights for developing effective therapies. Recent studies have shown that exosomes regulate human diseases by regulating immune responses, oxidative stress, autophagy, gut microbiome, and cell cycle (Baixauli et al., 2014; Muller et al., 2016; Real et al., 2018; Anel et al., 2019; Elashiry et al., 2020; Xu et al., 2020; Liu et al., 2021; Shen et al., 2021; Wang et al., 2021; Xing et al., 2021; Jahangiri et al., 2022; Yan et al., 2023). Exosomes form through the inward budding of early endosomal membranes (Figure 1), which mature into multivesicular bodies (MVBs). Multivesicular bodies are involved in endocytosis and transport of cellular material (Borges et al., 2013; Bebelman et al., 2018). Multivesicular bodies have two possible fates, including fusion with lysosomes, resulting in acidification and degradation of their contents, and fusion with the cytoplasmic membrane, releasing the endoluminal vesicles outside the cell and eventually forming exosomes (Raposo and Stoorvogel, 2013). Understanding the yield and protein composition of exosomes will improve our understanding of the molecular mechanisms underlying their formation. The exosomal formation mechanism involves two pathways, including the ESCRT (endosomal sorting complex required for transport) dependent pathway and the ESCRT independent pathway (Figure 2) (Dreyer and Baur, 2016). The ESCRT-dependent pathway comprises four protein complexes (ESCRT-0, -I, -II, -III), which regulate exosome formation and transport, with ESCRT-0 mediating substrate recognition and sorting, ESCRT-I and ESCRT-II mediating inward budding of endosomal membranes, and ESCRT-III shearing the bud neck to form MVB (Figure 3) (Hurley and Hanson, 2010; Hanson and Cashikar, 2012; Zeng et al., 2018).

Exosomes have been found in various body fluids, including blood, urine, cerebrospinal fluid, joint effusion, breast milk, and saliva (Nafar et al., 2022). Exosomes carry various molecules, including DNA, mRNA, non-coding RNA, lipids, cytokines, and proteins, and are crucial for cellular communication and information transfer (Mathieu et al., 2019). One previous study revealed that exosomes play an important role in modifying the extracellular microenvironment, immune regulation, homeostasis, and are incorporated into a variety of physiological and pathological processes (Raposo et al., 1996). Exosomes are mediators of intercellular communications as they deliver miRNA and other contents to recipient cells, and miRNA-containing exosomes can be absorbed by neighboring cells via endocytosis, thus influencing the phenotype of neighboring cells (Bruns et al., 2017). Exosomes can promote tumor progression and drug resistance in cancer by inhibiting the host immune response. Of note, exosomes released by tumor cells are implicated in resistance-associated secretory phenotype (RASP) by which immune escape is established. Heat shock proteins (HSPs) carried by exosomes can be co-transported with oncogenic factors to promote tumor development and escape to the immune system resulting in immunostimulatory effects (Taha et al., 2019). On the other hand, exosomes derived from platelets activated by thrombin may promote the survival, multiplication, and chemotaxis of hematopoietic cells as well as the release of pro-inflammatory cytokines (such as myeloperoxidase and superoxide dismutase) from monocytes, thus triggering local and systemic inflammation (Yasaka et al., 1981). Exosomes can be used as drug delivery vehicles as they are non-immunogenic, have good biocompatibility, and have efficient transport (Kim et al., 2018; Hou et al., 2019).

Exosomes have different biological characteristics and may exert different effects depending on their contents, tissue micro-environment, and recipient cells. Mature adipocyte-derived exosomes show a greater capacity for adipocyte differentiation than adipose stem cell-derived exosomes. Furthermore, adipocyte-derived exosomes can promote the differentiation of adipose-derived stem cells (ADSCs) (Dai et al., 2017). The repair and regenerative functions of adipose stem cell-derived exosomes mainly rely on the delivery of self-packaged proteins, lipids, mRNAs and miRNAs, and the required nutrients and inflammatory mediators (Cabral et al., 2018). Adipocyte-derived exosomes can promote insulin resistance and type II diabetes by affecting immune cells through paracrine pathways (Deng et al., 2009; Connolly et al., 2015; Ying et al., 2017). Studies have demonstrated that adipocyte-derived exosomes can influence foam cell formation and polarization, resulting in atherosclerosis (Kranendonk et al., 2014; Li X et al., 2019).

### 2.1 Negative regulation of adipocyte differentiation by exosomes

Wang et al. (2017) revealed that tumor-derived exosomes decrease the formation of lipid droplets and lower the mRNA expression of the adipogenic transcription factor, peroxisome proliferator-activated receptor γ (PPARγ, an adipocyte-specific marker), and lipoprotein lipase. They also demonstrated that pulmonary tumor-derived exosomes suppress adipogenic differentiation of ADSCs via the TGFβ signaling pathway.

### 2.2 Positive regulation of adipocyte differentiation by exosomes

Exosomes can induce their differentiation into mature adipocytes and cause lipid deposition (Wan et al., 2019). Adipocyte-derived exosomes (Ad-Exo) contain miRNA, RNA, and proteins that determine the lineage of human bone marrow mesenchymal stem cells (hMSCs). Ad-Exo can enhance ECM-induced differentiation of hMSCs to adipocytes. Narayanan revealed that the combination of osteoblast/adipocyte ECM and exosomes induced the expression of lineage-specific genes at the early differentiation stage. Their findings also showed that hMSCs differentiated on ECM of osteoblasts with adipogenic exosomes could express adipogenic genes (Narayanan et al., 2018).

Interfering ADSCs with adipose tissue-derived exosomes caused upregulated expression of PPARγ, adipocyte-specific fatty acid binding protein 2 (aP2), and lipocalin and an increase in the number of mature cells (Dai et al., 2017). However, exosomes released by lipopolysaccharide-activated macrophages did not affect the final maturation and differentiation of preadipocytes and fat storage (De Silva et al., 2018).

### 2.3 Regulation of lipid metabolism by adipose-derived exosomes

ADSCs-derived exosomes activate M2 macrophage polarization, express high levels of tyrosine hydroxylase, and enhance the expression of related thermogenic genes through a signal transduction and transcriptional activator 3-related pathway. Thus, they promote white adipose tissue browning, restore uncoupling protein 1 (UCP1)-dependent energy expenditure and ultimately reduce the effects of insulin resistance, dyslipidemia and hepatic steatosis caused by obesity (Zhao et al., 2018). A high expression of miRNA-155 in adipose tissue macrophages collected from obese mice effectively inhibit white fat browning and thermogenesis, reduce brown adipose tissue, as well as inhibits its function (Chen et al., 2013; Zhang et al., 2019).

A previous study showed a higher expression of Ad-Exo, in obese mice compared with non-obese mice (Lazar et al., 2016). Ad-Exo promotes fatty acid oxidation. Ad-Exo can increase the lipid content in adipose tissue macrophages (ATMs), and deliver triglycerides to macrophages, resulting in lipid accumulation in macrophages. Ad-Exo can induce the expression of lipid metabolism-related genes (Flaherty et al., 2019). Furthermore, mature adipocyte-derived exosomes are necessary for adipogenesis and rely on transient receptor potential mucolipin 1 (TRPML1) mediated lysosomal cytokinesis. Mature adipocyte-derived exosomes stimulate adipogenesis in a paracrine and autocrine manner. The expression of endogenous TRPML1 increased during the maturation and differentiation of preadipocytes. Notably, lipid synthesis reduced in the absence of TRPML1 (Kim et al., 2019).

In patients with obesity-related cardiovascular disease, Zhang found increased levels of cystatin C and CD14 (Zhang et al., 2016). Exosomes with high concentrations of cystatin C and CD14 have been linked to a higher risk of myocardial infarction and mortality. The expression of cystatin C in plasma exosomes was also positively associated with low-grade inflammatory response, low HDL-cholesterol levels and metabolic syndrome. Contrarily, a negative correlation was noted between the expression of CD14 and adipose tissue abundance, dyslipidemia, and a lower risk of type 2 diabetes (Zhang et al., 2016). Exosomes contribute to the onset of diabetes and its associated complications. Therefore, exosomes can act as novel therapeutic targets as well as biomarkers for early detection and staging of diabetes (Cianciaruso et al., 2017).

## 3 Regulation of adipogenic differentiation by exosome-derived ncRNAs

### 3.1 Regulation of adipogenic differentiation by miRNAs in exosomes

Mature miRNA can mediate mRNA degradation or suppress protein translation by binding with specific ribonucleoprotein AGO (Argonaute) to generate RNA-induced silencing complex (RISC). RISC recognizes target genes via complementary binding of miRNA seed sequences to mRNA 3′UTR or ORF regions (Bartel, 2004; Shenoy and Blelloch, 2014). miRNA can regulate mRNA expression by competitively binding to RNA-binding proteins (Wilczynska and Bushell, 2015). In the study by Son et al., it was found that miRNA can regulate adipogenesis by interacting with adipocyte differentiation-related transcription factor and key signaling molecules (Son et al., 2014). Table 1 shows various miRNAs and target genes that regulate adipocyte differentiation.

**TABLE 1:** The miRNAs associated with adipogenesis.

microRNA	Target	Function	Experiment model	References
miR-8	TCF	Proadipogenic	MSCs, ST2	Kennell et al. (2008)
miR-14	P38MAPK	Antiadipogenic	Adipose tissue	Xu et al. (2003)
miR-17-5p	BMPR2, BMP2	Proadipogenic	ADSCs	Li et al. (2013)
miR-17-92	Rb2/p130	Proadipogenic	3T3L1	Wang et al. (2008)
miR-21	TGFBR2	Proadipogenic	MSC, 3T3-L1, hASCs	Kim et al. (2009)
miR-22	HDAC6	Antiadipogenic	ADSCs	Huang et al. (2012)
miR-27a	PPARγ	Antiadipogenic	3T3L1	Kim et al. (2010)
miR-27b	PPARγ	Antiadipogenic	hMADS	Karbiener et al. (2009)
miR-29	AKT	Antiadipogenic	3T3L1	He et al. (2007)
miR-31	C/EBPα	Antiadipogenic	MSCs	Tang et al. (2009)
miR-33b	EBF1	Antiadipogenic	PSPA	Taniguchi et al. (2014)
miR-103	MEF2D	Proadipogenic	3T3L1	Li et al. (2015)
miR-106a	BMP2	Proadipogenic	ADSCs	Li et al. (2013)
miR-128	ABCA1, ABCG1	Proadipogenic	HEK293T, HepG2, MGF7	Adlakha et al. (2013)
miR-135a-5p	APC	Antiadipogenic	3T3L1	Chen et al. (2014a)
miR-137	CDC42	Antiadipogenic	ADSCs	Shin et al. (2014)
miR-138	EID-1	Antiadipogenic	MSCs	Yang et al. (2011)
miR-139-5p	NOTCH1, IRS1	Antiadipogenic	3T3L1	Mi et al. (2015)
miR-143	ERK5	Proadipogenic	3T3L1, MSCs	Esau et al. (2004); Oskowitz et al. (2008); Takanabe et al. (2008)
	PTN, ORP8	Glucose homeotatsis ↓	3T3L1, MSCs	Jordan et al. (2011); Yi et al. (2011)
miR-146b	SIRT1	Proadipogenic	3T3L1	Ahn et al. (2013)
miR-155	CREB	Antiadipogenic	3T3L1	Liu et al. (2011)
	C/EBPβ	Antiadipogenic	Adipose tissue	Liu et al. (2011)
miR-183	LRP6	Proadipogenic	3T3L1	Chen et al. (2014b)
miR-204-5p	DVL3	Proadipogenic	ADSCs	He et al. (2015)
miR-210	TCF7L2	Proadipogenic	3T3L1	Qin et al. (2010)
miR-224	EGR2, ACSL4	Antiadipogenic	3T3L1	Peng et al. (2013)
miR-302	CDKN1A	Proadipogenic	ASDCs	Kim et al. (2014)
miR-320	PI3K	Antiadipogenic	3T3L1	Ling et al. (2009)
miR-326	C/EBPα	Antiadipogenic	MSCs	Tang et al. (2009)
miR-335	MEST	Proadipogenic	3T3L1, MSCs	Zhu et al. (2014)
miR-363	E2F3	Antiadipogenic	ADSCs	Chen et al. (2014)
miR-375	ERK1/2	Proadipogenic	3T3L1	Ling et al. (2011)
miR-378a-3p	MAPK1	Proadipogenic	3T3L1	Huang et al. (2015)
miR-448	KLF5	Proadipogenic	3T3L1, MSCs	Kinoshita et al. (2010)
miR-486-5p	SIRT1	Antiadipogenic	ADSCs	Kim et al. (2012)
miR-540	PPARγ	Antiadipogenic	ADSCs	Chen et al. (2015a)
miR-548d-5p	PPARγ	Proadipogenic	hBMSCs	Sun et al. (2014)
miR-561	HSD11B1	Antiadipogenic	A549, HepG2	Han et al. (2013)
miR-579	HSD11B1	Antiadipogenic	A549, HepG2	Han et al. (2013)

Note: MAPK, mitogen-activated protein kinase; ERK, extracellular signal-regulated kinase; MSC, mesenchymal stem cell; PI3K, phosphoinositide 3-kinase; BMPR, bone morphogenetic protein receptor; HDAC, histone deacetylase; EBF1, early B-cell factor 1; MEF2, Myocyte Enhancer Factor 2; ABCA, ATP-binding cassette transporter A1; CDC, cell division cycle; NOTCH, neurogenic locus notch homolog protein; SIRT, silent information regulator; LRP, low density lipoprotein receptor related protein; DVL, dishevelled segment polarity protein; EGR, early growth response protein; CDKN, cyclin-dependent kinase inhibitor; PTN, pleiotrophin; ORP, oxysterol-binding protein-related protein; MEST, mesoderm specific transcript; HSD11b1, 11-Beta-Hydroxysteroid Dehydrogenase Type 1; cAMP, cyclic adenosine monophosphate; PKA, protein kinase A; CREB, cAMP, response element-binding; TCF, T-cell-specific transcription factor; TGF-β, transforming growth factor β; TGFBR2, TGF-β, receptor 2; hASC, human adipose tissue-derived stem cell; HMGA2, high mobility group AT-hook2; PPAR, peroxisome proliferator-activated receptor; hMADS, human multipotent adipose-derived stem; C/EBP, CCAAT/enhancerbinding protein; EID, EP300 interacting inhibitor of differentiation 1; KLF, Kruppel-like factor; BMSC, bone marrow stromal cell.

The three different types of adipocytes are referred to as white adipose tissue (WAT), brown adipose tissue (BAT), and beige adipose tissue. The WAT has increased white adipocytes. Mature white adipocytes contain a large lipid droplet that secrets some adipocytic factors, including leptin and adiponectin) (Cristancho and Lazar, 2011). Brown adipose tissue contains brown adipocytes. Classic brown adipocytes contain several small lipid droplets and mitochondria in the cytoplasm (Cristancho and Lazar, 2011). On the other hand, beige adipose tissue was discovered in recent years. Beige adipocytes develop in WAT under certain conditions, including long-term cold stimulation or following treatment with β3-adrenergic agonists (Harms and Seale, 2013).

PPARγ and C/EBPs are significant transcriptional regulators involved in adipocyte differentiation. miRNAs can interact with these transcription factors to regulate cell differentiation (Son et al., 2014). Adipose tissue macrophage-derived exosomes, miR-155 and miR-27, can prevent adipocyte differentiation by suppressing the expression of PPAR-γ. BAT mass decrease in mice upon overexpression of miR-155 (Chen et al., 2013). MiR-155 contained in milk exosomes may therefore reduce BAT differentiation. The miR-155 and miR-27 can regulate *in vivo* and *in vitro* insulin sensitivity (Ying et al., 2017). Furthermore, miR-27a and miR-130a can bind to the 3′UTR of PPARγ, resulting in the downregulation of PPARγ (Kang et al., 2013; Liu et al., 2021). The expression of miR-27a and miR-130a was downregulated during the differentiation of 3T3-L1. Upregulated expression of miR-27a and miR-130a is associated with downregulated expression of PPARγ and decreased adipocyte differentiation. In summary, miR-27a and miR-130a could inhibit adipocyte differentiation by targeting PPARγ. MiRNA-29b, another signature miR of cow’s milk exosomes, is also involved in adipogenesis (Bian et al., 2015). The C/EBPs transcription factor family is essential for the differentiation of adipocytes. C/EBPβ and C/EBPδ are induced early following exposure of preadipocytes to differentiation culture media, followed by induction of C/EBPα expression. C/EBPα acts as a transcriptional activator of multiple adipocyte genes and promotes adipocyte differentiation (Gregoire et al., 1998). The 3′-UTR of C/EBPα has two miR-326 binding sites (Feng et al., 2020). It has been reported that miR-326 binds to C/EBPα, thereby downregulating its expression and reducing adipogenic differentiation of hASCs. miR-31 can inhibit adipocyte differentiation by targeting C/EBPα, during differentiation of human mesenchymal stem cells (MSCs) into adipocytes (Sun et al., 2009; Tang et al., 2009).

miRNAs are involved in the regulation of adipocyte differentiation by the PI3K/Akt signaling pathway. In the PI3K/Akt signaling pathway, Akt is known as a downstream effector of c-Met. The miRNA-206 can prevent adipocyte differentiation by suppressing c-Met expression and reducing Akt phosphorylation (Tang et al., 2017). Additionally, by specifically targeting IRS1, miR-139-5p may have an adverse regulatory effect on adipocyte differentiation, which is a key member of the IRS1/PI3K/Akt insulin signaling pathway (Mi et al., 2015).

Adipocytes are derived from Mesenchymal stem cells (MSCs). MSCs first differentiate into adipoblasts, which then form precursor adipocytes that undergo clonal proliferation, growth arrest and terminal differentiation to form mature adipocytes. During the differentiation of MSCs into mature adipocytes, the pRB-E2F, MAPK, SMAD/TGFβ, WNT signaling pathways, and CCAAT/enhancer-binding proteins (C/EBPs) and peroxisome proliferator-activated receptors (PPRs) are activated. Esau discovered that the gene mitogen-activated protein kinase 5 (MAP2K5) is a target of miR-143, which can facilitate adipocyte differentiation (Esau et al., 2004). A previous study showed that miR-143 promoted adipocyte differentiation by inhibiting the expression of Pleiotrophin (PTN) (Yi et al., 2011). A study by Chen showed that transfection of miR-143 into hADSCs at different stages of differentiation induced varied effects (Chen et al., 2015b). Transfection during the mitotic proliferation phase prevented adipocyte differentiation, whereas transfection that occurred during the growth arrest or terminal differentiation stage facilitated differentiation. Additionally, miR-143 can regulate adipocyte differentiation by targeting the MAPK signaling pathway. Adipose tissues from obese people and mice given a high-fat diet had higher levels of exosome-derived miR-148a (Shi et al., 2015). miR-17-92 promotes adipocyte differentiation, whereas miR-363 inhibits adipocyte differentiation via the pRB-E2F signaling pathway (Wang et al., 2008; Chen et al., 2014). A previous study showed upregulated expression of miR-17-92 and downregulated expression of Rb2/P130 in hormone-stimulated 3T3L1 precursor adipocytes. The downregulated expression of Rb2/P130 caused reduced dimerization with the transcription factor E2F (E2F), thereby increasing free E2F4 and E2F5. Further, the free E2F4 and E2F5 activated the pRB-E2F signaling pathway, thus promoting the cells entry into the next cycle (Wang et al., 2008). Another study (Chen et al., 2014), exploring the miRNA expression profile of rat ADSCs during differentiation into mature adipocytes showed that miR-363 activated the retinoma-forming signaling pathway pRB-E2F by post-transcriptionally suppressing the translation of its target gene E2F3. This suppressed the expression of cyclin E (CYCE) and prevented the transition of cells from G1 to S phase, thereby inhibiting the clonal proliferation. The study showed downregulated expression of C/EBPα, which suppressed terminal cell differentiation.

Adipocyte differentiation is affected by miR-21, a miRNA that is a component of milk exosomes (Kim et al., 2012; Kang et al., 2013; Mei et al., 2013; Guglielmi et al., 2017). Kim showed that upregulated expression of miR-21 promoted adipogenic differentiation in hADSCs. In contrast, the inhibition of miR-21 suppressed adipogenic differentiation (Kim et al., 2009). The upregulation of miR-21 inhibited transforming growth factor beta receptor 2 (TGFβR2), which decreased the phosphorylation of Mothers against decapentaplegic homolog 3 (SMAD3), a downstream signaling molecule of the TGFβ/SMAD signaling pathway. miR-21 modulates the TGFβ/SMAD signaling pathway by inhibiting TGFβR2, a positive regulator of white adipocyte differentiation.

miRNAs can also bind to mRNAs of key molecules in signal transduction pathways and indirectly regulate adipocyte differentiation by modulating cell signaling. The Wnt/β-catenin signaling pathway is a key regulator of adipocyte differentiation. Activating Wnt pathway can inhibit adipocyte differentiation (Prestwich and Macdougald, 2007). miR-450a-5p promoted adipogenic differentiation of ADSCs in mice by targeting Wnt1-inducible signaling pathway protein 2 (WISP2) (Zhang et al., 2017). Chen et al. (2014) revealed that miR-344 could activate the Wnt pathway and inhibit adipocyte differentiation by binding to the 3′UTR region of GSK3-β (Glycogen synthase kinase 3-β), leading to the downregulation of GSK3-β expression and upregulation of β-catenin expression, the downstream effectors of GSK3-β. Chen showed that miR-183 promotes the differentiation of 3T3-L1 precursor adipocytes by targeting LRP6 (Low-density lipoprotein receptor-related protein 6), a Wnt signaling pathway molecule (Chen et al., 2014b).

A recent study showed that treating 3T3-L1 preadipocytes with TNF-α upregulated miR-155, which inhibited adipocyte differentiation by binding to the 3′UTR region of CREB (cAMP-response element binding protein) (Liu et al., 2011; Mi et al., 2015 also showed that miR-139-5p might prevent adipocyte differentiation by reducing Notch1 and IRS1 expression as well as preventing 3T3-L1 differentiation.

**FIGURE 4:**
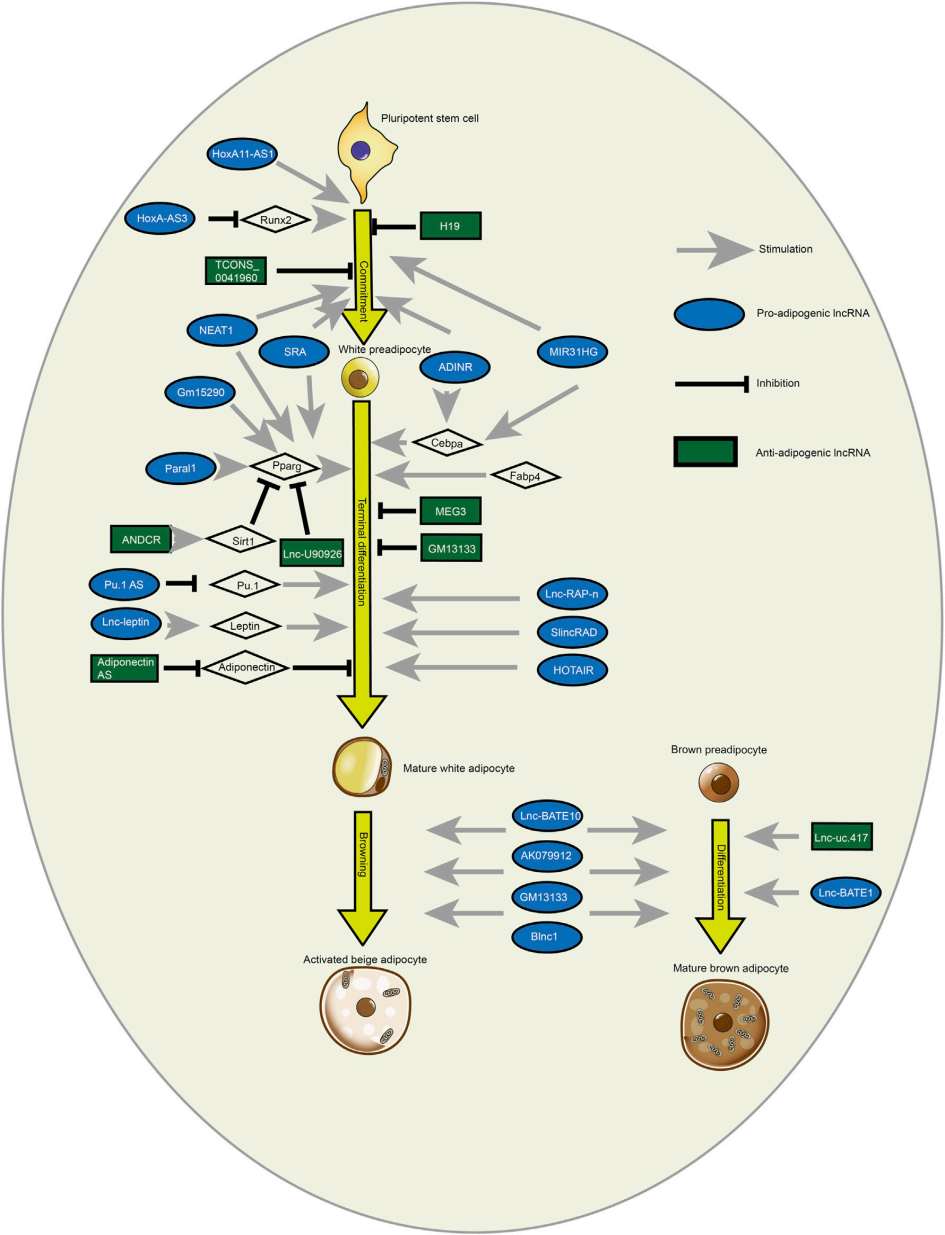
White and brown adipogenesis are affected by pro- and anti-adipogenic lncRNAs. LncRNAs regulate the fate of adipocytes derived from pluripotent stem cells. Brown adipocytes have many small lipid droplets and are abundant in mitochondrion compared to mature white adipocytes that contain TG in a solitary lipid droplet. Besides, LncRNAs modulate the browning of white adipocytes [Reprinted by permission from Elsevier Inc. Cellular signaling, Chen et al. (2018), copyright 2018].

The transcriptional activation of adipocyte-specific function genes is closely related to adipocyte differentiation. Multiple transcription factors are decisive in the differentiation of precursor adipocytes to mature adipocytes and gene transcription (Prestwich and MacDougald, 2007). PGC-1α, PRDM16, PPARγ and C/EBPs are essential transcription factors for brown adipogenesis (Figure 4) (Prestwich and MacDougald, 2007; Chen et al., 2018). miR-133 downregulation by cold stimulation was shown to promote brown adipocytes differentiation as miR-133 can downregulate PRDM16 expression by binding to its 3′UTR region (Trajkovski et al., 2012; Yin et al., 2013). Sun et al. demonstrated significantly upregulated expression of miR-193b-365 during brown adipocyte differentiation. miR-193b-365 can bind to myogenesis-related cytokines, Igfbp5 (Insulin-like growth factor binding protein 5) and Cdon to promote brown adipocyte differentiation and inhibit muscle formation (Sun et al., 2011).

miR-17-92 promotes preadipocyte differentiation by targeting Rb2/p130 mRNA. As preadipocytes undergo cell fusion and growth inhibition, the cell reenters the cell cycle under the stimulation of hormones. The cell begins to differentiate upon clonal expansion (Wang et al., 2008). In this process, cell transition from G1 to S phase depends on transcription factor E2F. The binding of Rb2/p130 to E2F protein inhibits the function of E2F, thereby promoting cell differentiation. miR-17-92 binds to Rb2/p130 mRNA and reduces the expression of Rb2/p130 protein by blocking its translation, thus promoting adipocyte differentiation. Additionally, miR-27a and miR-27b also inhibit adipocyte differentiation by targeting PPARγ expression (Kim et al., 2010).

Evidence show that miRNAs can regulate the browning of white adipocytes, thereby affecting beige fat formation (Fu et al., 2014; Hu et al., 2015). Lentivirus-mediated diet-induced downregulation of miR-34a in obese mice was shown to reduce blood lipid levels, increase mitochondrial copy number, and oxidative function in adipose tissue, as well as significantly increase the expression of beige fat marker proteins, CD137 and UCP-1 (Fu et al., 2014). miR-34a could increase the expression of fibroblast growth factor-1 receptor (FGFR1) by binding to its 3′UTR region. Downregulated expression of miR-34a was associated with upregulated expression of FGFR1 and enhanced signaling through the MAPK signaling pathway, causing increased phosphorylation of the extracellular regulated kinase (ERK) and increased beige fat formation. Downregulation of miR-34a can inhibit beige adipocyte differentiation by increasing PGC-1α deacetylation and improving the transcriptional activity of PGC-1α (Fu et al., 2014).

miR-122 is an abundantly expressed miRNA in the liver, and is engaged in the maintenance of liver homeostasis and lipid metabolism. Esau discovered that miR-122 deletion is associated with downregulation of several genes related to lipid metabolism, including acetyl-CoA carboxylase alpha (ACACA) and fatty acid synthase (FASN) (Esau et al., 2006). Additionally, Krützfeldt et al. (2005) found decreased plasma cholesterol levels in mice treated with antagomir-122. Overexpression of miR-122 was associated with increased transcription of several genes related to cholesterol biosynthesis and enhanced cholesterol synthesis. Exosomes from adipose tissue have considerably high levels of miR-122 (Huang et al., 2022), miR-103 (Li et al., 2015), miR-146b (Ahn et al., 2013), and miR-148a (He et al., 2018), which modulate adipogenesis.

MiRNA-33 is a crucial post-transcriptional regulator of intracellular cholesterol homeostasis and affects multiple pathways of lipid metabolism. miR-33 inhibits fatty acid β-oxidation by post-transcriptionally repressing the target genes, Carnitine O-octanoyltransferase (CROT), Trifunctional protein, beta subunit (HADHB) and Carnitine palmitoyltransferase 1A (CPT1A). The miR-33 regulates cholesterol efflux by targeting the ATP-binding cassette transporter A1 (ABCA1) (Esau et al., 2006; Iliopoulos et al., 2010). Also, miR-33 can inhibit negative regulation of SREBP proteins such as insulin receptor substrate 2 (IRS2), AMP-activated protein kinase alpha 1 subunit (AMPKA1) or AMP-activated protein kinase alpha 1 catalytic subunit (PRKAA1) and deacetylase 6 (SIRT6) (Ramirez et al., 2011). Cancer-associated cachexia patients exhibit elevated expression levels of miR-410-3p in their subcutaneous adipose tissues and serum exosomes, leading to significant suppression of adipogenesis and lipid synthesis (Sun et al., 2021). Exosomal miR-425-3p in tumor cells inhibit preadipocyte proliferation, prevent adipogenic differentiation, and promote adipocyte lipolysis and browning of white adipocytes (Liu et al., 2022). Furthermore, miR-27 (Ling et al., 2009; Yi et al., 2011; Zhu et al., 2014), miR-30c (Irani and Hussain, 2015), miR-122 (He et al., 2015), miR-144 (Ling et al., 2011), miR-168a (Zhang et al., 2012), miR-223 (Vickers et al., 2014a), miR-302a (Meiler et al., 2015), miR-370 (Iliopoulos et al., 2010), miR-378 (Gerin et al., 2010) and miR-758 (Ramirez, et al., 2011) can also exert regulatory effects on lipid metabolism.

### 3.2 LncRNA from exosome regulate adipogenic differentiation

To date, lncRNAs have been implicated in the regulation of various biological processes including growth and development, and other metabolic processes in the form of inducible, signaling, and scaffold molecules from epigenetic, transcriptional, and post-transcriptional regulation (Schmitz et al., 2016). Table 2 and Figure 2 present the lncRNAs which exert different functions during adipogenesis. The exosome acts as a transport mediator, encapsulating lncRNAs, and promoting their transit and function in target cells. Exosome-derived lncRNAs may influence adipocyte growth via pre- and post-transcriptional regulation of epigenetic regulation, competitive binding of miRNAs, and lipid metabolism by targeting inflammatory responses.

**TABLE 2:** The long noncoding RNA associated with adipogenesis.

LncRNA	Target	Function	Experiment model	References
HOTAIR	PPARγ	Proadipogenic	Primary preadipocytes from Human	Rinn et al. (2007); Gupta et al. (2010); Divoux et al. (2014)
ADINR	C/EBPα	Proadipogenic	Human MSCs	Xiao et al. (2015)
SRA	PPARγ	Proadipogenic	3T3L1	Xu et al. (2010); Liu S et al. (2014)
ADNCR	miRNA-204	Antiadipogenic	3T3L1, ADSCs	Li et al. (2016)
NEAT1	PPARγ2	Proadipogenic	3T3L1, ADSCs	Cooper et al. (2014); Gernapudi et al. (2016)
HoxA-AS3	Ezh2, Runx2	Proadipogenic	BMSCs	Zhu et al. (2016)
PU.1 AS	PU.1 mRNA	Proadipogenic	3T3L1	Pang et al. (2013)
SlincRAD	PPARγ	Proadipogenic	3T3L1	Yi et al. (2013)
Lnc RNA H19	miR-188	Antiadipogenic	BMSCs	Huang et al. (2016)
Lnc RAP-1	HnRNP-U	Proadipogenic	Primary predipocytes from mice	Quinn and Chang (2016)
U90926	PPARγ2	Antiadipogenic	3T3L1	Chen et al. (2017)
Blinc1	EBF2	Proadipogenic	Primary predipocytes from mice	Zhao et al. (2014), Mi et al. (2017)
Gm15290	miR-27b, PPARγ	Proadipogenic	Murine primary Preadipocytes, HEK293	Liu et al. (2017)
MIR31HG	Fabp4	Proadipogenic	ADSCs	Huang et al. (2017)
Paral1	RBM14	Proadipogenic	3T3L1	Firmin et al. (2017)
MEG3	miR-140-5p	Antiadipogenic	ADSCs	Li et al. (2017)
PVT1	PPARγ	Proadipogenic	3T3L1	Zhang et al. (2020)

Note: SRA, steroid receptor activator; NEAT1, nuclear-enriched abundant transcript 1; ADINR, adipogenic differentiation induced noncoding RNA; ADNCR, adipocyte differentiation-associated long noncoding RNA; Blnc1, brown fat enriched lncRNA, 1; ATGL, adipose triglyceride lipase; MSC, mesenchymal stem cell; PI3K, phosphoinositide 3-kinase; HOTAIR, HOX, transcript antisense intergenic RNA; BMPR, bone morphogenetic protein receptor; HDAC, histone deacetylase; EBF1, early B-cell factor 1; MEF2, Myocyte Enhancer Factor 2; ABCA, ATP-binding cassette transporter A1; EZH2, Enhancer of Zeste homolog 2; NOTCH, neurogenic locus notch homolog protein; SIRT, silent information regulator; LRP, low density lipoprotein receptor related protein; DVL, dishevelled segment polarity protein; EGR, early growth response protein; CDKN, cyclin-dependent kinase inhibitor; hnRNP, heterogeneous nuclear ribonucleoprotein; FABP4, Fatty Acid-Binding Protein 4; RBM14, RNA, Binding Motif Protein 14; PPAR, peroxisome proliferator-activated receptor; C/EBP, CCAAT/enhancer binding protein; BMSC, bone marrow stromal cell.

Gene imprinting is an epigenetic regulatory mechanism (Patten et al., 2014; Tucci et al., 2019), and lncRNA-H19 is a newly discovered lncRNA that inhibits adipogenic differentiation of BMSCs, and functions via epigenetic modification of histone deacetylases (Huang et al., 2016). lncRNA H19 overexpression inhibits obesity, improves insulin sensitivity, and promotes mitochondrial biosynthesis (Schmidt et al., 2018).

Insulin-like growth factor 2 (IGF 2) is an important imprinting gene, highly conserved in vertebrates (Smits et al., 2008), and is closely related to individual lean meat percentage, backfat thickness as well as other productive properties. Methylation occurs in the ICR region, one of the key pathways responsible for controlling gene imprinting. Additionally, exosome lncRNA may affect the adipogenic capacity of an individual by modulating IGF2 expression, thereby disrupting lipid accumulation (Hark et al., 2000; Smits et al., 2008).

Recent studies have shown that exosome lncRNA competitively binds with miRNA and circRNA to co-regulate gene expression (Jalali et al., 2013), ensuring a stable expression of lipid metabolism-related target genes. The sponge effect of lncRNA has now been used to bind miRNA in order to analyze its molecular structure and regulatory mechanism (Tong et al., 2019; Yao et al., 2019). Additionally, MALAT1 is linked to the occurrence of metabolic diseases and is involved in lipid metabolism in the liver (Liu JY et al., 2014; Yan et al., 2014).

Silencing MALAT1 in ob/ob mice abolishes the palmitate-induced increase in nuclear SREBP-1c protein and hepatic lipid accumulation by inhibiting stearoyl-Coenzyme A desaturase 1 (SCD1), FAS, Acetyl-CoA carboxylase 1 (ACC1) and ATP citrate lyase (ACLY), which are the major targets of SREBP-1c (Lee et al., 2013). MALAT1 may enhance lipid accumulation via a mechanism involving contact with SREBP-1c to increase the level of nuclear SREBP-1c protein. Notably, lncHR1 is implicated in the transcriptional inhibition of SREBP-1c expression (Li D et al., 2017). Similar to lncHR1, lncRNA Gm16551 suppresses SREBP1c in the mouse liver (Yang et al., 2016). Gm16551 in the liver has been found to inhibit lipid biosynthesis. The increased triglyceride circulation and adipogenic gene expression caused by SREBP1c are inhibited by Gm16551 overexpression, which increases the possibility that Gm16551 might interfere with SREBP1c functions.

Peroxisome proliferators-activated receptors (PPARs) can regulate lipid metabolism, including subtypes PPAR α, β, and γ. Among them, PPARγ is primarily expressed in the immune system and modulates glucose and lipid metabolism. Ligand of PPARγ not only improves insulin resistance and blood lipid, immune regulation and anti-inflammation, but also induce anti-tumor cell proliferation and promotes cell differentiation. It has been reported to alter various biological behaviors of tumor cells such as metastasis and invasion. By influencing the signaling pathway regulated by NFκB and activator protein-1, PPARγ can effectively inhibit the activation and transcription of target gene promoters, achieving its intended purpose. Genes containing peroxisome proliferator responsive element (PPRE) structure include caproyl coenzyme A synthetase, lipoprotein lipase (LPL), insulin receptor substrate-2 (IRS-2), leptin and tumor necrosis factor-α (TNF-α) (Lapsys et al., 2000). PPARγ is related to the incidence and progression of multiple diseases, including diabetes, obesity, and hypertension. Specifically, PPARγ is a key factor in adipocyte differentiation and has attracted much attention in recent years.

Steroid receptor RNA activator (SRA) was the first lncRNA implicated in adipogenesis to be discovered (Xu et al., 2010). By binding to PPARγ, SRA in adipose tissue increases its transcriptional activity and promotes the differentiation of 3T3-L1 preadipocytes. It can regulate the adipocyte cell cycle, insulin-related signaling pathway, and TNF-α signaling pathway (Xu et al., 2010). In the liver, SRA can also promote lipid metabolism via promoter activity suppression of adipose triglyceride lipase (ATGL) by targeting transcription factor FoxO1, thereby downregulating ATGL expression to promote hepatic steatosis (Chen et al., 2016). During the maturation and differentiation of preadipocytes, NEAT1 expression is upregulated, and its activation is necessary for adipogenesis; this suggests that it is a positive regulator of lipogenic differentiation (Pang et al., 2013). Cooper et al. (2014) reported that lncRNA NEAT1 expression temporally fluctuates during the differentiation of 3T3-L1 adipocytes into adipocytes and modulates multiple splicing of PPARγ mRNA, a major transcription factor in adipogenesis. Similarly, other lncRNAs, U90926, Paral1, Gm15290, and Plnc1, also regulate adipogenesis by targeting key adipogenesis transcription factor PPARγ (Chen et al., 2017; Firmin et al., 2017; Liu et al., 2017; Zhu et al., 2019).

LncRNA U90926 is a class of adipose tissue-specific lncRNAs whose expression is downregulated with 3T3-L1 differentiation, its overexpression reduces 3T3-L1 adipogenic differentiation capacity, whereas its knockdown has the opposite effect (Chen et al., 2017). lncRNA-U90926 knockdown in 3T3-L1 cells inhibits the transcriptional activity of the PPARγ promoter, thereby suppressing 3T3-L1preadipocyte differentiation (Chen et al., 2017). Gm15290 overexpression significantly promotes adipocyte differentiation, whereas its interference significantly suppresses adipocyte differentiation; lnc RNA Gm15290 upregulates PPARγ expression level to promote adipogenic, thereby affecting lipid accumulation in the mouse model (Liu et al., 2017). Firmin et al. (2017) found upregulated Paral1 expression during adipogenic differentiation, which increased the transcription activity of PPARγ by synergistically activating RNA binding motif protein 14 (RBM14). In turn, PPARγ upregulated Paral1 expression and its binding level to RBM14. This feedback loop mechanism promotes adipogenesis.

LncRNA IMFNCR promotes adipogenic differentiation of adipocytes in muscle by regulating PPARγ expression through competitively repressing miRNA-128-3p and miRNA-27b-3p (Khalil et al., 2009). Of note, lncRNA-GAS5 is an important gene that regulates adipocyte proliferation and growth. GAS5 can regulate the expression level of cAMP via PDE4B and function on DNL-related enzymes (e.g., ACC1, FAS) and those related to mitochondrial function (e.g., PGC-1α) through phosphorylation of CREB downstream of Camp; thus, this enhances lipid *de novo* synthesis, inhibits mitochondrial oxidative function, and eventually promotes lipid accumulation in hepatocytes (Xu et al., 2022). The lncRNA GAS5 suppresses the inhibitory effect of miR-18a on connective tissue growth factor (CTGF), thereby reducing adipogenic differentiation of MSCs (Li M et al., 2018).

Adipocyte differentiation-associated long noncoding RNA (ADINR) is a newly discovered ncRNA that regulates adipogenesis (Xiao et al., 2021). ADINR could specifically bind to PA1 during adipocyte differentiation, recruiting methylates lysine and MLL3/4 histone methyltransferase complex, increasing H3K4 (histone H3 lysine4) methylation levels at the C/EBPα site, promoting C/EBPα expression and accelerating adipogenesis. Labeled genes related to adipocyte differentiation were downregulated after ADINR silencing, resulting in reduced lipid accumulation, and suppressed adipogenic differentiation (Xiao et al., 2021).

ADNCR is a lncRNA that is reported to be significantly downregulated during adipocyte adipogenic differentiation (Li et al., 2016). ADNCR, as a competing endogenous RNA (ceRNA) of miR-204, can dependently and competitively upregulate the expression of a target gene, a silent information regulator of transcription 1 (SIRT1), suppressing adipocyte differentiation.

Adiponectin AS is another negative regulator is, whose expression is upregulated during adipose differentiation and plays a negative regulatory role. It forms RNA double-stranded aggregates during the translocation from the nucleus to the cytoplasm, which inhibit the translation of AS mRNA and cause a reduction of body weight, adipose tissue content, and hepatic triglycerides in DIO (diet-induced-obese) mice, thus acting as a barrier in adipogenic differentiation (Cai et al., 2018).

HOTAIR, a lncRNA is involved in preadipocyte differentiation and can promote adipocyte differentiation in abdominal preadipocytes (Divoux et al., 2014). When HoxA-AS3 expression is suppressed in bone marrow mesenchymal stem cells (BMSCs), the formation of fat and fat marker gene expression decreases, implying that HoxA-AS3 is important in adipogenesis (Zhu et al., 2016). LncRNA MIR31HG decreases the enrichment of active histone markers H3K4me3 and AcH3 in the promoter of adipogenesis-related genes FABP4, thereby inhibiting its expression and adipogenesis (Huang et al., 2017).

### 3.3 Regulation of adipogenic differentiation by circRNAs in exosomes

circRNA is a type of RNA with a covalent closed-loop structure; circRNA from adipose tissue exosomes can function in an autocrine pathway (Shah et al., 2018). circRNA, which is enriched in exosomes, is important for inter-cell communication (Shang et al., 2020). Due to the absence of a 5′ or 3′ end, circRNA is resistant to nuclease-mediated degradation and is thought to be more stable than linear RNA (Salzman et al., 2012). Current studies on the mechanism of exosome circRNA focus on its capacity to effectively act as a miRNA sponge (Zhang et al., 2020; Zhang et al., 2020). Since circRNA is abundant in miRNA binding sites, it can reduce the repressive effect of miRNA on target genes and increase the expression level of those genes via competitive binding to miRNA (Ebert et al., 2007). circRNA can also regulate pre-mRNA by targeting alternative splicing or transcription (Ashwal-Fluss et al., 2014). Table 3 presents the circular RNAs which exert different functions during adipogenesis.

**TABLE 3:** The circular RNA associated with adipogenesis.

circRNA	Origin	Target	Function	References
circARF3	Adipose tissue (mice)	miR-103	Alleviate adipose inflammation	Zhang et al. (2019)
circNrxn2	Adipose tissue (mice)	miR-103	Promotes WAT browning	Zhang et al. (2019)
circRNA_26852	Adipose tissue (pig)	ssc-miR-486, ssc-miR-874	Regulates adipogenic differentiation	Li A et al. (2018)
circSAMD4A	Visceral adipose tissue	miR-138-5p	Regulates preadipocytes differentiation	Liu et al. (2020)
circFUT10	Adipose tissue (bovine)	miR-let-7c	Inhibits adipocyte differentiation	Jiang et al. (2020)
hsa-circRNA9227-1	HPA-v/adipocytes	hsa-miR-665	Regulates adipogenesis	Sun et al. (2020)
circRNA_0046367	HepG cells	miR-34a, PPARα	Regulates lipid metabolism	Guo et al. (2017a)
circScd1	NAFLD mouse	JAK2/STAT5 pathway	Regulates lipid metabolism	Li P et al. (2019)

Note: circARF3, ADP-ribosylation factor 3; circSAMD4A, sterile alpha motif domain containing 4A; BMI, body mass index; HPA-v, human preadipocytes from visceral fat tissue; ITIH5, inter-alpha-trypsin inhibitor heavy chain; LPL, lipoprotein lipase; JAK, janus protein tyrosine kinases; STAT, signal transducer and activator of transcription; NAFLD, nonalcoholic fatty liver disease; WAT, white adipose tissue.

circH19 secreted by hADSCs can bind to polypyrimidine tract-binding protein 1 (PTBP1), suppressing the translocation of sterol regulatory element-binding protein 1 (SREBP1) from the cytoplasm to the nucleus, consequently inhibiting cell differentiation and lipid accumulation (Park et al., 2012; Oliva-Olivera et al., 2016; Zhu et al., 2020). Adipose tissue exosome circRNA realizes its function through paracrine. Patients with obesity often have a slower rate of wound healing, than healthy individuals, and circRNA Circ_0075932, which is a single exon exosome secreted by his/her adipose tissues, can bind to pumilio homolog 2 (PUM2), promoting the expression of serine/threonine protein kinase, activating nuclear factor kappa beta (NF-kB) pathway to induce inflammation and apoptosis, eventually accelerating healing (Zhang X et al., 2019).

Circ FUT10 competitively represses the expression of target gene let-7c and can inhibit adipogenic differentiation in adipocytes by disrupting the expression of its target genes PPARγ coactivator-1α (PGC1α) (Jiang et al., 2020). On the other hand, circ SAMD4A promotes adipocyte differentiation by acting as a “sponge” for miR-138-5p, thereby upregulating the expression of enhancer of zeste homologue 2 (EZH2), whereas adipocyte differentiation becomes significantly inhibited after circ SAMD4A knockdown (Liu et al., 2020).

In high-fat diet (HFD)-induced obese mice, mmu_circ_0000529 (the homologous mouse circRNA for circSAMD4A) knockdown alleviated weigh gain, lowered food intake, reduced body fat, and increased energy consumption. Additionally, it improved insulin sensitivity and glucose tolerance (Liu et al., 2020). *In vitro* tests demonstrated that circSAMD4A can bind to miR-138-5p and function as a miRNA sponge to subsequently regulate the expression of EZH2 (Liu et al., 2020). circSAMD4A was significantly upregulated in obese people compared to lean people, and its expression level was associated with a poor prognosis for obese patients (Liu et al., 2020). Functional analysis revealed that circSAMD4A overexpression can regulate preadipocyte differentiation and accurately forecast the development of obesity in humans.

Exosomal plasma from osteoporotic patients demonstrated a higher expression level of has_circ_0006859 than that from healthy individuals, and has_circ_0006859 can act by inhibiting miR-431-5p expression, subsequent upregulation of rock1 (rho-associated coiled-coil containing protein kinase 1), as well as increasing the probability of hBMSCs to differentiate into adipocytes rather than osteoblast (Zhi et al., 2021). Guo et al. (2017b) constructed a regulation network of circRNA-miRNA-mRNA to analyze the mechanism of metabolism by applying high-fat stimulation to build HepG2 fatty liver. Consequently, circRNA_021412 and miR-1972 showed weakened inhibition on LPIN1. LPIN1 induced down-expression of Long-chain-fatty-acid-CoA ligase (ACSL), thus resulting in fatty liver. This evidence confirms that circRNA is a key regulatory factor in fatty liver, and transcription-dependent regulation in metabolic pathways is partially caused by circRNA_021412/miR-1972/LPIN1 signal.

During *in vivo* and *in vitro* steatosis of hepatocytes, the normalized expression of circRNA-0046367 can facilitate PPARs to return to normal expression, furtherly improving lipid metabolism disorder, thereby effectively preventing hepatocytes steatosis from lipid peroxidation (Guo et al., 2017a). Therefore, circRNA-0046367 is a potential therapeutic intervention for speroxidative damage.

Accumulating evidence suggests that circRNAs are strongly connected to non-alcoholic fatty liver disease (NAFLD), a condition characterized by a variety of factors including hepatic lipid accumulation, insulin resistance (IR), adipose tissue, mitochondrial dysfunction, a high-fat diet, obesity, a chronic inflammatory state (Huang et al., 2017; Liu et al., 2017).

circRNA genome-wide abnormal regulation is responsible for hepatocytes steatosis (Guo et al., 2017b). For instance, CircScd1 downregulation could damage Janus kinase2/signal transducer and activator of the transcription 5 pathway, causing fatty steatosis in NAFLD (Li P et al., 2019). circRNA_021412/miR-1972/LPIN1 signal might be a key factor in circRNA-related fatty acid metabolic disorder, resulting in increased adipogenesis. Meanwhile, circRNA_0046366 could help normalize the PPAR signal to suppress hepatocyte fat steatosis. Bioinformatic and functional analyses revealed that circRNA_0046366 is a miR-34a-specific antagonist. Because of its multi-targeting characteristics, miR-34 promotes an antagonistic effect on adipose-related miRNA/mRNA interactions based on competitive binding (Li et al., 2011; Dekkers et al., 2018; Gulsin et al., 2018; Lee et al., 2018). As the only target of circRNA_0046366, miR-34a modulates the lipid metabolism of hepatocytes. circRNA_0046366 expression suppresses the level of hepatocytes TG, and circRNA_0046366 is important in hepatocyte fat steatosis. Disruption of the circRNA_0046366/miR-34a/PPARα signal may be a new epigenetic basis of hepatocyte fat steatosis, suggesting that circRNA_0046366 is a potential treatment target for hepatocytes steatosis (Guo et al., 2018). Abnormal genomic regulation of circRNA is often thought to cause hepatocytes steatosis (Dekkers et al., 2018). Transcriptional regulation is thought to be involved in the metabolism pathway. circRNA_021412/miR-1972/LPIN1 signal may be the key factor affecting circRNA-related fatty acid metabolic disorder, resulting in adipogenesis. As indicated, circTshz2-1 and circArhgap5-2 are key adipogenic regulators *in vitro*. Also, *in vivo* silencing of circArhgap5-2 inhibits lipid accumulation and downregulation of adipogenesis markers, suggesting that circArhgap5-2 is important to preserving lipid biosynthesis and metabolism in the whole transcription process of adipocytes (Arcinas et al., 2019).

## 4 Role of exosomal ncRNAs in diseases

**FIGURE 5:**
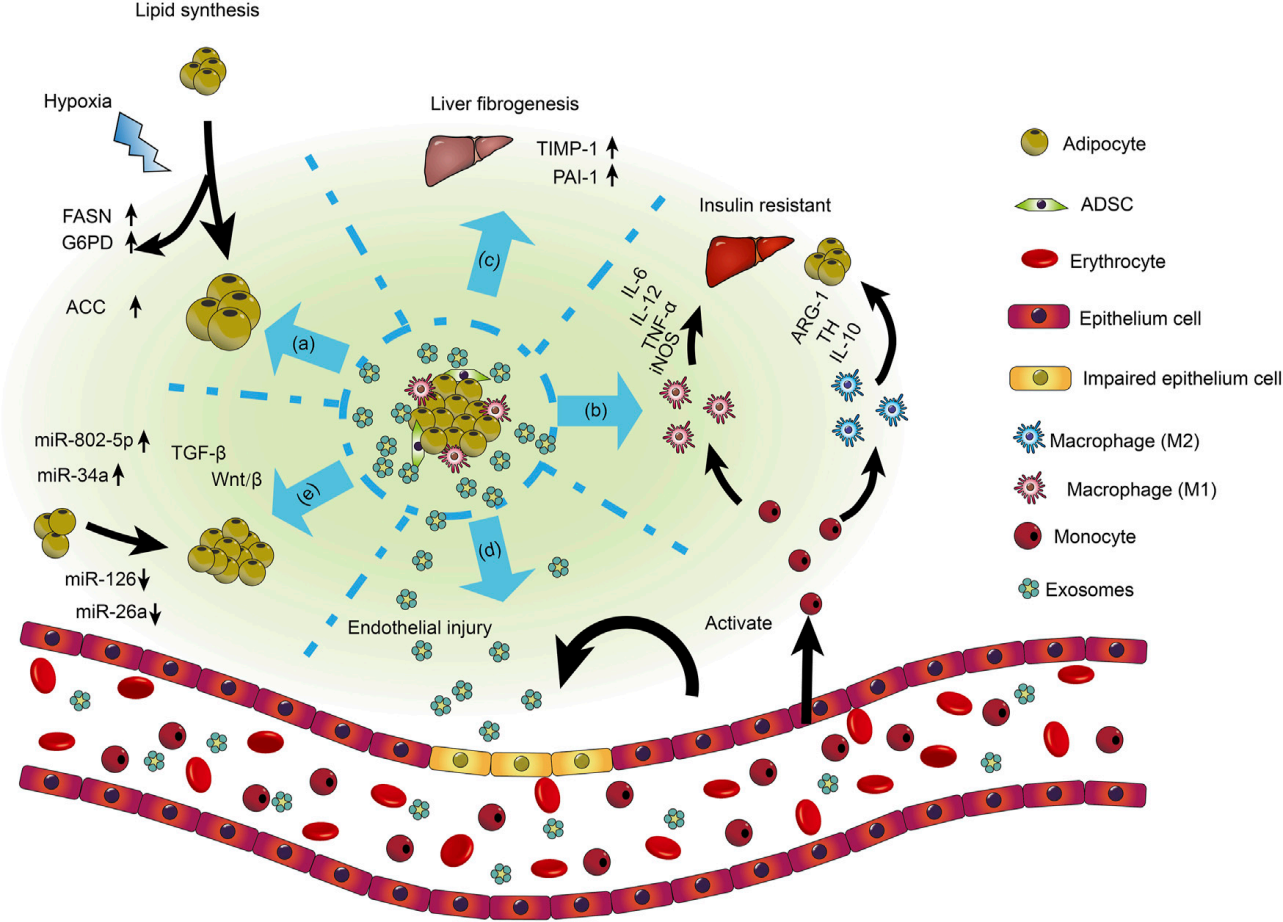
Roles of exosomes in obesity-related disorders. MSC-derived exosomes reduce insulin resistance by promoting M2 macrophage polarization and repressing M1 macrophage polarization. Lipid droplets enlarge after ingestion by tiny adipocytes from exosomes. Insulin resistance in adipocytes and liver develops as a result of exosomes from the adipose tissue of obese people activating monocyte differentiation into macrophages and triggering the release of inflammatory factors. Exosomes released from dysfunctional and hypertrophic adipocytes can impair the functional ability of vascular endothelial cells. Exosomes produced from adipose tissue contain miRNA, necessary for modulating gene expression in adipose tissue and other cells. Adipose tissue-derived exosomes that contain various miRNAs are partially upregulated, while others are downregulated [Reprinted by permission from John Wiley and Sons Ltd. Cell Proliferation, Zhang et al. (2016), copyright 2016].

As newly discovered means in inter-cellular communications, exosomes modulate the progression of different human diseases (e.g., cancer, metabolic diseases). They comprise growth factors, protein, lipids, DNA, miRNA, lncRNA, and circRNAs (Li C et al., 2021). Once released to the extracellular environment, exosomes bind to receptors and deliver carried ncRNAs, inducing functional reactions and phenotype changes (Figure 5) (Zhang et al., 2016). Exosomal analyses focus on how they regulate inter-cellular signal transduction, immune system function, functional development, and differentiation in the tumor microenvironment. Additionally, the application of exosomal ncRNAs is continually expanding on clinical diagnosis and treatment.

### 4.1 Exosomal ncRNAs and insulin resistance

IR is among the crucial sources of Type 2 Diabetes Mellitus (T2DM) (Qiu et al., 2018). It contributes to the development of Alzheimer’s disease and gestational diabetes mellitus (GDM). Insulin resistance refers to insulin-induced tissue damage, such as facilitating glucose absorption and inhibiting glycogen metabolism in adipocytes and skeletal muscle cells. Besides its role in glucose metabolism, insulin also affects the metabolism of proteins and lipids (Qiu et al., 2018). A knockout of insulin β-cell exosome-derived β linc1 (β-cellintergenic non-coding RNA1) gene causes insulin secretion disorder in adult mice and can impair islet cell maturation as well as differentiation in embryonic mice (Arnes et al., 2016).

Adipose tissues play a significant role in insulin resistance, and exosomes derived from adipose tissue may mediate this process. Exosomes secreted by the adipose tissues of mice can induce macrophage cell activation, where retinol-binding protein 4 (RBP4) is the key activation factor (Deng et al., 2009). Additionally, the exosomes linked to obesity deliver macrophages to the liver and adipose tissues, where macrophages release TNF-α and IL-6, causing insulin resistance. Song et al. (2018) uncovered that insulin-resistant adipocyte-derived exosomes (IRADE) treated macrophages could promote insulin resistance in adipose tissues by lowering insulin receptor substrate-1 (IRS-1) and hormone-sensitive lipase (HSL) expression.

Active substances in exosomes modulate inter-cellular insulin signal transduction. Pancreatic β-cells secrete miR-29 family members (miR-29s) via exosomes to regulate glucose homeostasis by manipulating glucose output in the liver, thereby inhibiting insulin signaling in the liver, whereas blocking miR-29s expression in pancreatic β-cells can reverse (HFD)-induced insulin resistance (Li J et al., 2021). Adipose tissue macrophages (ATM) of mice with obesity can secrete exosomes transferring exosomes with miRNA into insulin target cells, which can evaluate FFA levels in the blood, damaging insulin sensitivity, and enhancing insulin resistance (Ying et al., 2017). Additional studies have confirmed that exosomes from ATM of normal mice can reduce insulin resistance in obese mice, whereas exosomes with miR-15 from ATM of obese mice can cause insulin resistance (Ying et al., 2017).

Adipocyte-derived exosomes can promote macrophage activation by accelerating M1 polarization and inhibiting M2 polarization, before activating insulin resistance. Serum exosomes from patients with T2DM and insulin resistance exhibit markedly upregulated miR-222 expression levels. High miR-222 expression in mouse adipose tissue-derived exosomes can aggravate insulin resistance in the liver and skeletal muscle of obese mice on a high-fat diet by suppressing insulin receptor substrate 1 (IRS1) expression (Li et al., 2020).

As endocrine factors, ADSC-derived exosomes (ADSC-Exos) can disrupt the metabolism of corresponding target organs and functions by biologically active vesicular molecules. In the liver, miRNAs carried by ADEVs could regulate PPARγ and FGF-21 expression and are involved in glucose tolerance and insulin sensitivity regulation (Thomou et al., 2017). In cases of obesity, additional organs may also generate IR-related exosomes. miR-130a-3p-containing exosomes secreted by the liver can improve glucose tolerance by inhibiting PH domain leucine-rich phosphatase 2 (PHLPP2) expression in adipocytes; On the other hand, in the case of specific knockdown of miR-130a-3p, mice with obesity showed a notable increase in blood glucose level as well as a decrease in glucose tolerance and insulin sensitivity *in vivo* (Wu et al., 2020). This indicates that miRNAs secreted by hepatic exosomes can significantly reduce insulin resistance. Pancreatic islets are involved in the regulation of insulin sensitivity and can act also produce insulin. For instance, miR-26a from pancreatic β-cell could improve insulin sensitivity with preserved β-cell function.

miR-27a and miR-320 expressions in plasma exosomes are noticeably higher in T2DM patients than in healthy individuals, making it possible for exosomes with specific bioactive substances to act as serum biomarkers of T2DM (Zhang et al., 2018). Notably, the Dicer enzyme is an RNA endonuclease; Thomou et al. (2017) used Cre-Lox recombination to construct a Dicer-KO mouse. In contrast with wild mice, the quantity of white adipose tissues in the experimental mice was reduced, causing insulin resistance, adipose tissue inflammation, and dyslipidemia; consequently, miRNA expression in its circulating exosomes becomes significantly downregulated. Transplanting adipocyte-derived exosomes with multiple miRNAs, particularly miR-99b, can restore circulating miRNA, reducing FGF21mRNA expression and its 3′-UTR receptor activity, eventually regulating lipid metabolism in multiple tissues (Thomou et al., 2017). Considering the molecular regulatory mechanism, in which lncRNA can function for the competitive binding of ceRNA to miRNA, it is hypothesized that exosome-derived lncRNA may activate pro-inflammatory factors via its abnormal expression, potentially causing abnormal glucolipid metabolism including insulin resistance or alteration of glucose tolerance in the body.

## 5 Exosomal ncRNAs as a diagnostic biomarker of human diseases

Biomarkers are molecules used for disease detection and/or prognostic prediction. An effective biomarker exhibits high sensitivity and specificity, good stability, and anti-interference. Exosomes are broadly spread in different bodily fluids with various sources and can easily be utilized and stably detected. Various human diseases possess various expression levels of exosomal ncRNAs, including cancers and metabolic diseases, laying a foundation for ncRNAs as a biomarker in early diagnosis.

Abnormal expression of exosome-derived lncRNA is responsible for disordered lipid metabolism (Khalil et al., 2009; Yin et al., 2013; Zhu et al., 2019). Exosomes are characterized by targeted delivery and can protect lncRNA from interferences from exogenous substances. As a result, lncRNA can be effectively maintained in target cells and function normally. Thus, analysis of the regulation of lipid metabolism through the perspective of exosomes may reveal important biomakers. Future studies should attempt to predict fat deposition sites in livestock and even regulate lipid accumulation.

With continuing research on the function of exosomal lncRNA molecular markers, there is increasing evidence on the application of exosome-derived lncRNAs as biomarkers to indirectly regulate lipid metabolism. Koeck et al. (2014) isolated exosomes from the adipose tissue of patients with obesity, and co-cultured it with hepatocytes *in vitro*. As previously stated, when exosomes are transported into hepatocytes, the expression of enzymes, including TIMP-1 and other bio-active factors, is altered, causing a disorder in the NAFLD related transforming growth factor β (TGFβ) pathway, and ultimately lipid metabolism disorder.

lncRNA has been shown to affect NAFLD symptom formation (Chi et al., 2021). Researchers analyzed the expression pattern of lncRNA-MEG3, as well as its regulatory function on triglyceride and adipogenesis-related genes, using free fatty acids induced HepG2 cells and HFD-mice to establish biological models *in vitro* and *in vivo* (Chi et al., 2021). Downregulation of lncRNA-MEG3 suppresses adipogenesis-related genes, whereas MEG3 over-expression reduces lipid accumulation in cells. The hidden molecular mechanism could be that lncRNA-MEG3 competitively binds to miR-21 with LRP6 to suppress the mTOR pathway and stimulate lipid accumulation in cells, resulting in NAFLD. The results demonstrated that exosome-derived lncRNA-MEG3 might be used as a NAFLD biomarker.

Exosomes can be used as detection markers and treatment targets to alleviate insulin resistance. A useful test that reveals the general physical state is liquid biopsy. Because of their sensitivity to changes in human psychological and pathological states, exosomes have the potential to act as a biomarker in insulin resistance. Exosomes have a membrane structure that prevents inner molecular degeneration, allowing their use in detecting nuclear acid changes in patients. With a deeper understanding of exosomes, we will learn more about their benefit as a liquid biopsy tool, particularly in early diagnosis of insulin resistance, where exosome is the most promising liquid biopsy method. Notably, potential exosomal biomarkers, including Let-7b, miR-144-5p, and miR-34a carried by exosomes in plasma (Jones et al., 2017) and miR-20b-5p carried by exosomes in serum (Katayama et al., 2019), can all be candidates in diagnosing insulin resistance. Exosomes regulate the incidence and development of diabetes and its associated complications, and thus it can be used as an early detection and staging biomarker of diabetes mellitus, and as promising treatment targets in diabetes.

## 6 Disease treatment based on exosomal ncRNAs (metabolic diseases and obesity/diabetes)

Exosome, a potential biomarker and treatment target, is a clinically promising carrier for therapeutic agents. Exosomes, ncRNAs mimics, and inhibitors can be prepared through separation and purification, followed by exosome surface modification to improve target specificity, then used as a carrier to transport ncRNAs to target tissue or organ. However, there is no standardized protocol for the processing and characterization of exosomes, and further studies are needed to address this issue (Lai et al., 2022). Advances in biomedical materials and molecular targeted therapy may also help uncover the role of additional exosomal ncRNAs.

Exosomes have also shown an advantage in disease diagnosis, and the ncRNAs stored inside are closely associated with disease progression, acting as a novel biomarker for diseases such as diabetes. Nevertheless, the interaction network between bioactive molecules in exosomes and their receptors remains unclear. Research on exosomes as a novel diagnostic and treatment tool remains in its infancy and warrants substantial efforts before clinical application.

## 7 Conclusion and outlook

Exosomal ncRNAs have a high potential as a biomarker in multiple human diseases and therapeutic medicine. However, with a focus on early disease diagnosis markers, exosome suppression, and drug delivery, ncRNAs carried by exosomes have recently provided novel ideas and methods in function and related mechanisms of adipogenesis, development and energy metabolism to prevent obesity. Nevertheless, research on exosomes and the lipid metabolism pathway is currently limited. Thus, to ensure that exosomes are a direct source of relevant biomarkers to broaden the source of the exosome remain unresolved. The pharmacokinetics, pharmacodynamics, and toxicity of exosomal ncRNAs remain to be clinically validated. Also, the regulation of exosomal ncRNAs in psychological and pathological process warrant additional analysis. Because adipose tissues interact with other organs or tissues of the body (central nervous system, important metabolic organs, and immune system), the dynamics of adipocyte lipid droplets and regulation of lipid metabolism by external nutrients, signals, and stress states merit additional investigation and exploration. Research on exosome origin, synthesis and secretion, intake, and other mechanisms is necessary to accelerate the development of diagnostic and prognostic biomarkers of disease.
